# Inflammation Controls Susceptibility of Immune-Experienced Mice to Sepsis

**DOI:** 10.4049/immunohorizons.2200050

**Published:** 2022-07-25

**Authors:** Roger R. Berton, Isaac J. Jensen, John T. Harty, Thomas S. Griffith, Vladimir P. Badovinac

**Affiliations:** * Department of Pathology, University of Iowa, Iowa City, IA;; † Interdisciplinary Program in Immunology, University of Iowa, Iowa City, IA;; ‡ Department of Microbiology and Immunology, Columbia University, New York, NY;; § Department of Urology, University of Minnesota, Minneapolis, MN;; ¶ Minneapolis VA Health Care System, Minneapolis, MN

## Abstract

Sepsis, an amplified immune response to systemic infection that leads to life-threatening organ dysfunction, affects >125,000 people/day worldwide with 20% mortality. Modest therapeutic progress for sepsis has been made, in part because of the lack of therapeutic translatability between mouse-based experimental models and humans. One potential reason for this difference stems from the extensive use of immunologically naive specific pathogen-free mice in preclinical research. To address this issue, we used sequential infections with well-defined BSL-2 pathogens to establish a novel immune-experienced mouse model (specific pathogen experienced [SPexp]) to determine the extent to which immunological experience and/or inflammation influences the host capacity to respond to subsequent infections, including sepsis. Consistent with their immunological experience, SPexp inbred or outbred mice had significant changes in the composition and activation status of multiple leukocyte populations known to influence the severity of cecal ligation and puncture–induced sepsis. Importantly, by varying the timing of sepsis induction, we found the level of basal inflammation controls sepsis-induced morbidity and mortality in SPexp mice. In addition, although a beneficial role of NK cells in sepsis was recently demonstrated in specific pathogen-free mice, NK cell depletion before cecal ligation and puncture induction in SPexp mice lead to diminished mortality, suggesting NK cells may have beneficial or detrimental roles in the response to septic insult dependent on host immune status. Thus, data highlight the importance of utilizing immune-experienced models for preclinical studies to interrogate the cellular/molecular mechanism(s) that could be therapeutically exploited during severe and dysregulated infection-induced inflammatory responses, such as sepsis. *ImmunoHorizons*, 2022, 6: 528–542.

## INTRODUCTION

Sepsis is an amplified immune response to systemic infection, leading to life-threatening organ dysfunction, characterized by an initial transient cytokine storm composed of proinflammatory and anti-inflammatory cytokines. Once the cytokine storm subsides, a chronic state of immune dysfunction, termed immunoparalysis, develops and can persist for months to years (1, 2). Current clinical data suggest nine people develop sepsis every 6 s globally, and two of those people succumb as a result of the overactive immune system and poorly controlled cytokine storm (3). Although the sepsis mortality rate has decreased in recent decades, this improvement reflects more on the significant advancements in critical care, with substantial effort and little progress (>100 failed phase II and III clinical trials) made in modulating the cytokine storm itself (4). A major unresolved issue in the field is the impact that “baseline” level of inflammation has on the acute immune response during a septic event. For example, TNF-α neutralization enhances survival in LPS-challenged specific pathogen-free (SPfree) mice, but this potential therapy failed to improve survival in laboratory mice born to wild mice or translate to improvements in septic patients (5, 6). Lack of therapeutic translatability between mice and humans is thought to arise, in part, from the extensive use of SPfree mice in preclinical sepsis research.

SPfree mice have been instrumental in our understanding of immunology and human disease because of their experimental reproducibility, ease of genetic manipulation, and highly refined systems of immunological interrogation (e.g., adoptive cell transfer) (7–10). Although mice and humans share >90% genetic homology (7, 11) the human immune system is shaped by daily exposure to pathogenic and commensal microbes, creating an immunologically experienced host—something that is not observed in SPfree mice. Thus, an adult SPfree mouse is immunologically more similar to a neonatal human, rather than adult and elderly humans (a substantial patient cohort susceptible to sepsis or other infections) (12–14). Thus, it is pertinent to understand how we can better bridge the immunological gap between mice and humans. One approach to mimic the complexity of immunological experience in adult humans is the use of “dirty” mice, generated through natural pathogen transfer that results in a host that immunologically resembles adult humans (7, 12, 14, 15). Notably, we recently demonstrated these dirty mice have increased susceptibility to a sepsis-induced cytokine storm (16). As such, altered responses in dirty mice highlight the importance of utilizing immunologically experienced murine models in translational research.

Although dirty mice are closing the gap between mice and humans, their utilization comes at an experimental and fiscal cost. When generating dirty laboratory mice by cohousing with pet store mice, the laboratory mice receive a continual undefined bolus of pathogens resulting in variable cohorts with prolonged inflammation (9, 12, 16). Cohousing SPfree mice with pet store mice can also lead to significant mortality (12), prompting the need for increased numbers of mice that increase experimental costs. Lastly, the utilization of dirty mice may not be achievable at all institutions because of additional institution-specific requirements (e.g., BSL-3 housing) used to prevent the spread of unknown pathogens in conventional facilities (7). As an alternative to the dirty mouse models using pet store/wild mice and special housing, we developed an immunologically experienced murine model (specific pathogen experienced [SPexp]) using sequential infections with well-studied BSL-2 laboratory pathogens (i.e., influenza A virus [IAV], CMV, vaccinia virus, attenuated *Listeria monocytogenes*, and lymphocytic choriomeningitis virus–Armstrong [LCMV-Arm]). This infection strategy drives the expansion and maturation of multiple adaptive and innate immune cell populations to promote the interrogation of how immune experience results in altered responses to subsequent challenges. The generation of SPexp mice through controlled sequential infections enables fine-tuning of the level of “experience” and inflammation before interrogating how this immunological experience influences susceptibility to subsequent challenge, including sepsis. The sequential infection model we developed is similar to the one described by Reese et al. (13), who showed sequential infection generates immunologically experienced mice exhibiting immune traits seen in adult PBMCs. Finally, the use of known pathogens allows us to draw conclusions based on published data specific to each pathogen.

In this study, we demonstrate that sequential infection of mice led to a transient increase in inflammation and TLR expression and a long-term altered cellular composition and activation status of multiple leukocyte populations. In addition, the level of inflammation (or cytokines/chemokines produced) and TLR expression contributes to the rate of mortality after a septic event. Lastly, in contrast with SPfree hosts, NK cells exacerbate septic mortality in SPexp hosts. These results have direct implications for preclinical interrogation of therapeutic intervention during the cytokine storm.

## MATERIALS AND METHODS

### Ethics statement

Experimental procedures using SPfree and sequentially infected mice were approved by University of Iowa Animal Care and Use Committee under Animal Care and Use Review, protocol #9101915, and housed under BSL-1 and BSL-2 conditions, respectively. Experimental procedures using SPfree mice cohoused (CoH) with pet store mice were approved by the University of Minnesota Animal Care and Use Committee, protocol #1906–37113A, and housed under BSL-3 conditions. All experiments followed Office of Laboratory Animal Welfare guidelines and Public Health Service Policy on Human Care and Use of Laboratory Animals. Experimental animals were humanely euthanized by cervical dislocation.

### Mice and infections

**FIGURE 1.**
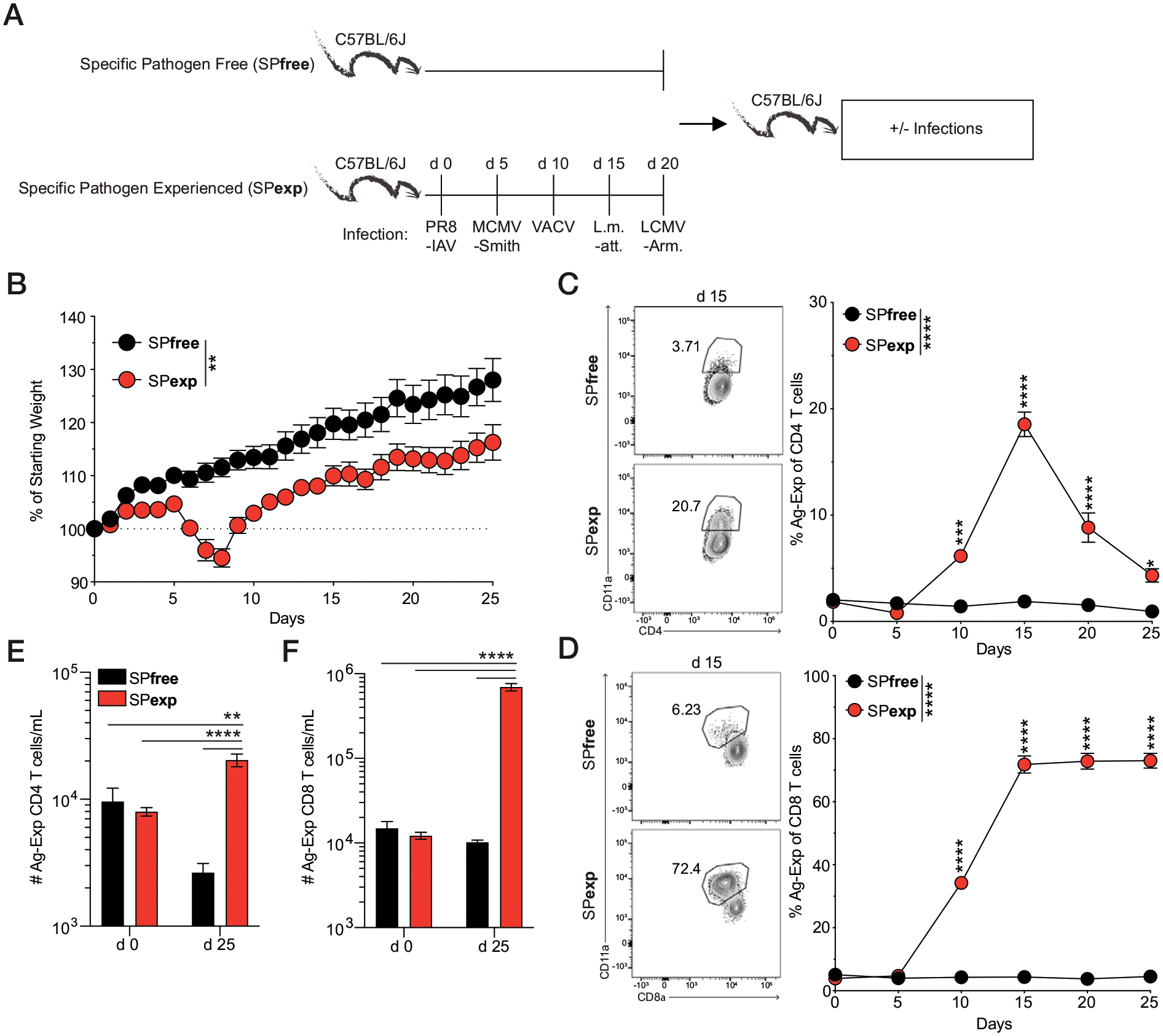
Frequencies of Ag-experienced CD4 and CD8 T cells during the generation of SPexp mice. (**A**) Experimental design. Sequential infection of SPfree C57BL/6 mice with PR8, MCMV-Smith, vaccinia virus (VACV), attenuated *L. monocytogenes* (L.m.-att), and LCMV-Arm in 5-d intervals was used to generate SPexp mice. The frequency of Ag-experienced CD4 and CD8 T cells (CD3^+^CD4^+^CD11a^hi^ and CD3^+^CD8^lo^CD11a^hi^, respectively) in the PBLs was monitored every 5 d for 25 d. (**B**) Body weight changes were monitored during SPexp generation. Longitudinal analysis and representative gating (day 25 after PR8) of Ag-experienced (**C**) CD4 and (**D**) CD8 T cells. Prior gating on CD3^+^ single-cell lymphocytes. Numbers of Ag-experienced (**E**) CD4 and (**F**) CD8 T cells on days 0 and 25 after PR8 infection. Data are representative of at least three independent experiments with 5–10 mice per group. Error bars represent SEM. *p < 0.05, **p < 0.01, ***p < 0.001, ****p < 0.0001.

Inbred C57BL/6 mice (wild-type, Thy1.2/1.2) were bred at the University of Iowa, and outbred Swiss Webster mice were purchased from Charles River. Mice were maintained in the animal facilities at the University of Iowa at the appropriate biosafety level. Both male and female mice >6 wk old were used in experiments, yielding similar results in both sexes. Generation of SPexp mice was achieved through sequential infection of SPfree mice with 10^3^ PFUs IAV/Puerto Rico/8/34 (PR8; H1N1) intranasally (17), followed by 10^5^ PFUs murine CMV (MCMV)-Smith (or MCMV-K181) i.p. (18), 10^6^ PFUs vaccinia virus i.p. (19), 10^7^ CFUs attenuated *L. monocytogenes* (strain DPL1942) i.v. (20), and 2 × 10^5^ PFUs LCMV-Arm i.p. (21) in 5-d intervals (Fig. 1A). Age-matched SPfree mice served as controls. In indicated experiments, SPfree and SPexp (without *L. monocytogenes* infection) mice were challenged with 8 × 10^4^ CFUs virulent *L. monocytogenes* (strain 10403s) i.v. (16) at indicated days after LCMV-Arm infection.

### Cell isolation, fluorescent labeling, and flow cytometric analysis

Peripheral blood was collected retro-orbitally from isoflurane-anesthetized mice. The blood was treated with ACK lysis buffer for 5 min to remove RBCs, leaving the peripheral blood leukocytes (PBLs). To determine expression of cell surface molecules, we incubated PBLs with mAb at 4°C for 20–30 min, and cells were subsequently fixed for 10 min using Cytofix Solution (BD Biosciences). The following mAb clones were used to stain processed samples: CD11a (M17/4; eBioscience), TLR4 (SA15-21; BioLegend), CD3ε (145-2C11; BioLegend), NK1.1 (PK136; eBioscience), NKp46 (29A1.4; BioLegend), F4/80 (BM8; BioLegend), TLR2 (CB225; BD Bioscience), CD19 (6D5; BioLegend), CD11c (HL3; BD Biosciences), CD4 (GK1.5; BioLegend), Ly6G (1A8; BioLegend), CD127 (A7R34; BioLegend), Ly6C (HK1.4; BioLegend), and CD8α (53-6.7; BioLegend). Flow cytometry data were acquired on a Cytek Aurora (Cytek, Bethesda, MD) and analyzed with FlowJo software (Tree Star, Ashland, OR).

### Cytokine analysis

Multiplex cytokine analysis was performed via Bio-Rad Laboratories Bio-plex Pro Mouse Cytokine 23-Plex according to the manufacturer’s instructions for plasma cytokine analysis. Samples were analyzed on a Bio-Rad Laboratories Bio-Plex (Luminex 200) analyzer in the University of Iowa Flow Cytometry Core Facility.

### Cecal ligation and puncture model of sepsis

Sepsis was induced by cecal ligation and puncture (CLP) (22) at indicated times after LCMV-Arm infection or in age-matched SPfree mice. In brief, mice were anesthetized with ketamine/xylazine, the abdomen was disinfected with Betadine (Purdue Products), and a midline incision was made. Thereafter, the distal third of the cecum was ligated with Perma-Hand Silk (Ethicon), punctured once (for moderate septic insult with ~10–20% mortality [CLP_20_]) or twice (for severe septic insult with ~50% mortality [CLP_50_]) using a 25-gauge needle (23), and a small amount of fecal matter was extruded out of each puncture. The cecum was then returned to the abdomen, and the peritoneum was closed with 641G Perma-Hand Silk (Ethicon). Bupivacaine (Hospira) was then administered at incision site, and skin was closed using surgical Vetbond (3M). Directly after surgery, 1 ml of PBS was administered s.c. to provide after surgery fluid resuscitation, and flunixin meglumine (Phoenix) was administered for postoperative analgesia. Sham mice underwent identical laparotomy surgical procedures, excluding CLP.

### Disease severity evaluation

Clinical signs of disease were evaluated and used for scoring disease severity. Clinical scores were assessed by ascending morbidity scale (24)—grooming: 0, (normal); 1, fur that has lost shine/become matte (dusty); 2, fur becomes erect or bristling (ruffled); mobility: 0, mobile without stimulation (normal); 1, mice are less responsive/mobile to stimuli (reduced); 2, mice are unresponsive to stimuli (immobile); body position: 0, body is fully extended (normal); 1, back is arched/curved (hunched); 2, laying on side at rest (on side); weight loss: 0, due to minimal weight loss that occurs in Sham controls weight loss has been adjusted, to allow for surgery-specific weight loss to be mitigated (<10%); 1, moderate weight loss (10–15%); 2, severe weight loss (>15%). After giving one score for each category, the sum of all categories indicates disease score. Importantly, dead mice are given the highest score (8) on the day of death and thereafter removed from scoring. Healthy scores range from 0 to 2, moderate disease scores range from 3 to 5, and severe disease scores range from 6 to 8.

### Generation of microbially experienced ‘dirty’ mice and cytokine measurements

Female C57BL/6N mice were purchased from Charles River (Wilmington, MA) at 8–10 wk of age. Female pet store mice were purchased from local pet stores in the Minneapolis-St. Paul metropolitan area. All mice were housed in American Association of Laboratory Animal Care–approved BSL-3 animal facilities at the University of Minnesota. SPfree B6 and pet store mice were CoH at a ratio of 8:1 in large rat cages for 60 d to facilitate microbe transfer (25). Blood was collected from the SPfree B6 mice before cohousing and at weeks 2, 4, 6, and 8 of cohousing. Serum was separated from the clotted blood, and the concentrations of IP-10 (CXCL10), IFN-γ, IL-1β, IL-6, IL-10, and TNF-α were quantitated with a custom Procarta-Plex assay using a Luminex 200 with Bio-plex Manager Software 5.0 at the University of Minnesota Cytokine Reference Laboratory.

### NK cell depletion

NK cell depletion was performed as previously described (26). In brief, mice were depleted of NK cells by administering 300 μg of anti-NK1.1–depleting Ab i.p. at indicated times. Control mice were given the same amount of rat IgG. Administration of Abs on days of surgery was done immediately after surgery (abdominal closure).

### Statistical analysis

Data were analyzed with Prism 9 GraphPad software and Microsoft Excel software for serum radial graphs. Summary data generated are represented as mean ± SEM. Violin plots and bar graphs were analyzed using unpaired *t* test (if unequal variance, Mann–Whitney *U* test was used), stacked bar graphs and morbidity curves with two-way ANOVA with Bonferroni test, and Kaplan–Meier survival curves with log-rank (Mantel–Cox) tests (*p < 0.05, **p < 0.01, ***p < 0.001, ****p < 0.0001).

## RESULTS

### Sequential infection increases inflammation and alters cellular composition

In contrast with adult humans, >6-wk-old SPfree B6 mice contain low frequencies and numbers of Ag-experienced T cells (12, 27, 28), a key feature of an immunologically experienced host. Importantly, immunological experience can be achieved through natural pathogenic and commensal microbe transfer, as shown in laboratory mice CoH with pet store mice, implanting embryos into wild-caught mice, or maintaining mice in enclosed outdoor areas (5, 15, 16, 29, 30). Given the complexities of these housing conditions (e.g., need for BSL-3 housing, wild mice, outdoor enclosures, and institutional approval to introduce mice carrying a multitude of microbes normally excluded from standard SPfree housing), we developed a sequential infection model to generate SPexp mice (Fig. 1A, with arrow indicating abbreviated experimental scheme). Specifically, we sequentially infected SPfree B6 mice with the commonly used experimental pathogens IAV-PR8, MCMV-Smith, vaccinia virus, attenuated *L. monocytogenes*, and LCMV-Arm in 5-d intervals via prototypical infection route (17–21). Our familiarity with using these pathogens to stimulate multiple Ag-specific T cell populations, as well as being able to house these mice under BSL-2 conditions, served as justification for this model (18, 31–35). Twenty-five days after PR8-IAV infection (and 5 d after last infection with LCMV-Arm), SPexp mice have increased body weight compared with their starting weight (Fig. 1B), suggesting these mice are not chronically ill. Moreover, the doses used of IAV, *L. monocytogenes*, vaccinia, and LCMV-Arm result in acute infections because the mouse immune system can clear these pathogens within 7–10 d (17, 36, 37). MCMV-Smith, by contrast, establishes a latent infection (38). Together, this combination of bacterial and viral pathogens serves as a reproducible model of acute and chronic infection that may occur in humans.

**FIGURE 2.**
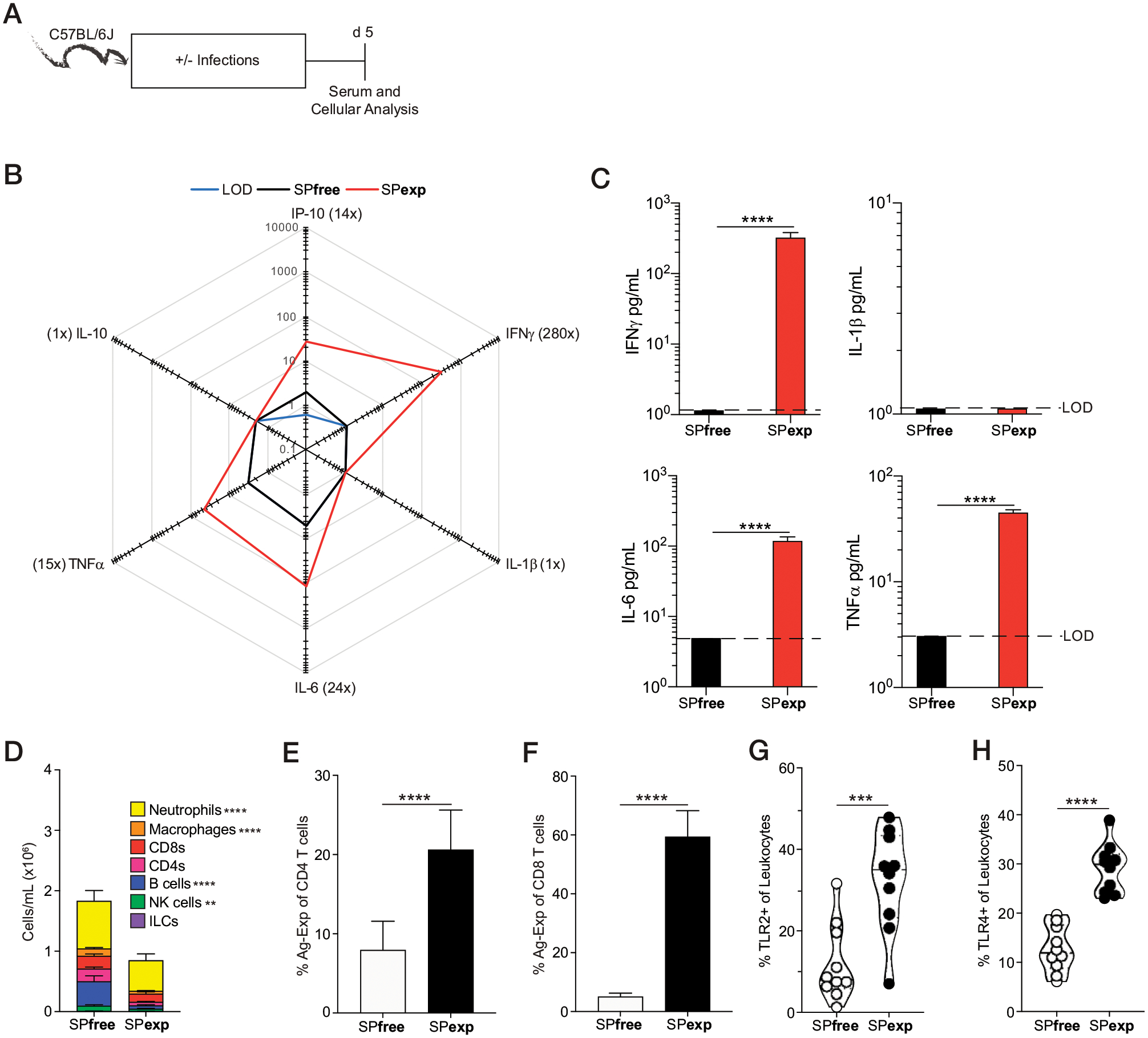
Sequential infection leads to increased inflammation and alters cellular composition and phenotype. (**A**) Experimental design. Serum and PBLs were collected from SPexp and age-matched SPfree B6 mice 5 d after last infection. (**B**) Summary data of analyte levels 5 d after last infection. Concentrations are in pg/ml. Parentheses indicate fold difference between experimental groups. (**C**) IFN-γ, IL-1β, IL-6, and TNF-α concentrations in SPfree and SPexp mice 5 d after last infection. (**D**–**H**) Number of cells per milliliter of PBL (D), frequency of Ag-experienced (E) CD4 and (F) CD8 T cells, and frequency of (G) TLR2- and (H) TLR4-expressing leukocytes 5 d after last infection. Data are representative of at least three independent experiments with 10 mice per group. Error bars represent SEM. **p < 0.01, ***p < 0.001, ****p < 0.0001.

As expected, sequential infection with these pathogens increased the frequency of Ag-experienced CD4 and CD8 T cells by day 10 after the first infection (IAV), and these frequencies remained elevated 5 d after the last infection (LCMV-Arm, day 25; Fig. 1C, 1D). Importantly, the numbers of Ag-experienced CD4 and CD8 T cells were significantly increased in SPexp 25 d after initial infection in the blood (Fig. 1E, 1F). To further characterize the immunological changes that occur after sequential infection, we bled SPexp and age-matched SPfree mice at day 5 after last infection to define the cellular and cytokine composition (Fig. 2A). Sequential infection significantly increased the concentration of proinflammatory cytokines IFN-γ, IL-6, and TNF-α, which were ≥280, 24, and 15 times higher than that seen in SPfree mice, respectively (Fig. 2B, 2C), clearly demonstrating that sequential infection with common experimental pathogens elevates the systemic inflammatory response. Interestingly, SPexp mice had decreased total cellularity within the PBLs, which was accompanied by decreases in neutrophils, macrophages, B cells, and NK cells (Fig. 2D). Notably, the observed cellular decrease in SPexp mice may be because of cell death or migration to infected tissues, which requires further interrogation. SPexp mice, as expected, had increased frequencies of circulating Ag-experienced CD4 and CD8 T cells 5 d after the last infection (Fig. 2E, 2F). Laboratory mice CoH with pet store mice have increased TLR2 and TLR4 expression (16), and this increased expression contributes to increased or decreased susceptibility to subsequent challenges relative to immunologically naive controls. To determine the extent to which TLR2 and TLR4 expression are modulated in SPexp mice, TLR2- and TLR4-expressing leukocytes were assessed in the blood at day 5 after last infection. Notably, SPexp mice had increased frequencies of TLR2^+^ and TLR4^+^ leukocytes, consistent with observations in microbially experienced laboratory mice CoH with pet store mice (16), cumulatively demonstrating that sequential infection alters the activation status of multiple cell types (Fig. 2G, 2H).

Although microbial exposure-induced immune experience is different between conventionally housed naive mice and humans, another difference is genetic diversity because a majority of preclinical experiments are performed using inbred mouse strains, such as C57BL/6 (7, 39, 40). Therefore, SPexp outbred Swiss Webster mice were generated to determine whether genetically diverse hosts undergo similar cellular immunological changes after sequential infections (Supplemental Fig. 1A). Similar to their inbred counterparts, outbred SPexp mice had increased proinflammatory cytokines (IL-2, IL-6, TNF-α, and IFN-γ), decreased cellularity in the blood, and an increased frequency of Ag-experienced CD4 and CD8 T cells within PBL 5 d after last infection (Supplemental Fig. 1B–E), as seen in inbred SPexp mice (Fig. 2). In addition, sequential infections in outbred SPexp mice resulted in an increased prevalence of TLR2- and TLR4-expressing leukocytes (Supplemental Fig. 1F, 1G). Taken together, exposing inbred and outbred mice to sequential infections with well-defined BSL-2 experimental pathogens in a relatively short time frame (20+ days) generated immunologically experienced hosts characterized by increased inflammation and changes in the composition and activation status of multiple leukocyte populations relative to immunologically naive SPfree mice.

### Immunological experience achieved by sequential infection alters host response to newly introduced infections

**FIGURE 3.**
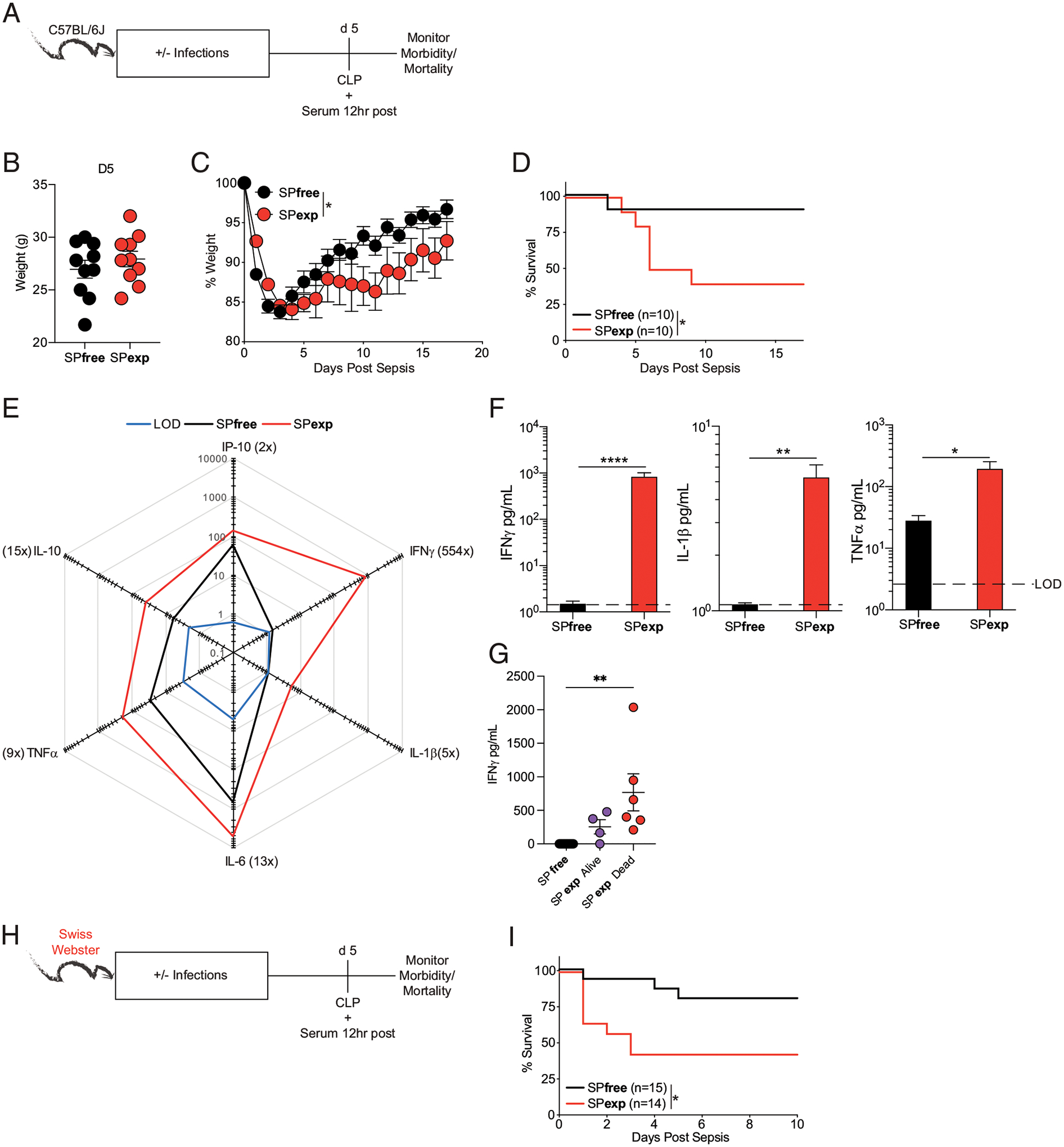
Immunologically experienced mice have exacerbated responses to sepsis. (**A**) Experimental design. Moderate septic event (<20% mortality in SPfree mice) was induced via CLP 5 d after last infection in SPexp and age-matched SPfree B6 mice. Weight loss and mortality were monitored over time, and serum cytokines were measured 12 h after CLP. Weight of mice (**B**) before and (**C**) after CLP. (**D**) Mortality of mice after CLP. (**E**) Summary data of analyte concentrations (pg/ml) in mice 12 h after CLP. (**F**) Individual serum IFN-γ, IL-1β, and TNF-α concentrations between mice 12 h after CLP. (**G**) Individual serum IFN-γ concentrations between SPfree and SPexp mice that survived or succumbed to CLP surgery. Data are representative of at least three independent experiments with 10 mice per group. (**H**) Experimental design. Five days after last infection, CLP was induced in SPfree and SPexp Swiss Webster mice. (**I**) Survival curves after CLP. Data are representative of at least three independent experiments with 10–15 mice per group. Error bars represent SEM. *p < 0.05, **p < 0.01, ****p < 0.0001.

Microbially experienced CoH mice have altered responses to newly introduced infections compared with SPfree mice, including enhanced clearance of virulent *L. monocytogenes*, but decreased survival after polymicrobial sepsis (16). The potential for generalized microbial exposure to have either beneficial or detrimental consequences on subsequent microbial challenge prompted us to examine the magnitude of the systemic cytokine storm in SPexp mice after CLP-induced sepsis, as well as monitor morbidity and mortality, relative to age-matched SPfree mice (Fig. 3A). Although SPfree and SPexp mice had similar weights before surgery and lost similar weight after CLP, SPexp mice exhibited delayed weight recovery and enhanced mortality after the septic event (Fig. 3B–D). These findings were recapitulated in outbred SPexp mice, which had increased mortality when CLP was performed 5 d after the last infection with LCMV-Arm (Fig. 3H, 3I). Importantly, the enhanced morbidity and mortality in inbred SPexp mice was accompanied by increased proinflammatory and anti-inflammatory cytokines 12 h after CLP (Fig. 3E, 3F). Therefore, similar to the findings observed in CoH ‘dirty’ mice; the sepsis-induced cytokine storm and susceptibility (mortality) to septic insult are increased in SPexp mice compared with SPfree mice. The notion that the magnitude of basal inflammation present at the time of sepsis induction predetermines the outcome/susceptibility to CLP surgery was supported by serum IFN-γ concentrations before surgery. A trending increase in the concentration of IFN-γ was observed in SPexp mice that succumbed compared with the mice that survived CLP surgery (Fig. 3G), suggesting the extent of inflammation present at the time of sepsis induction influences susceptibility to sepsis.

**FIGURE 4.**
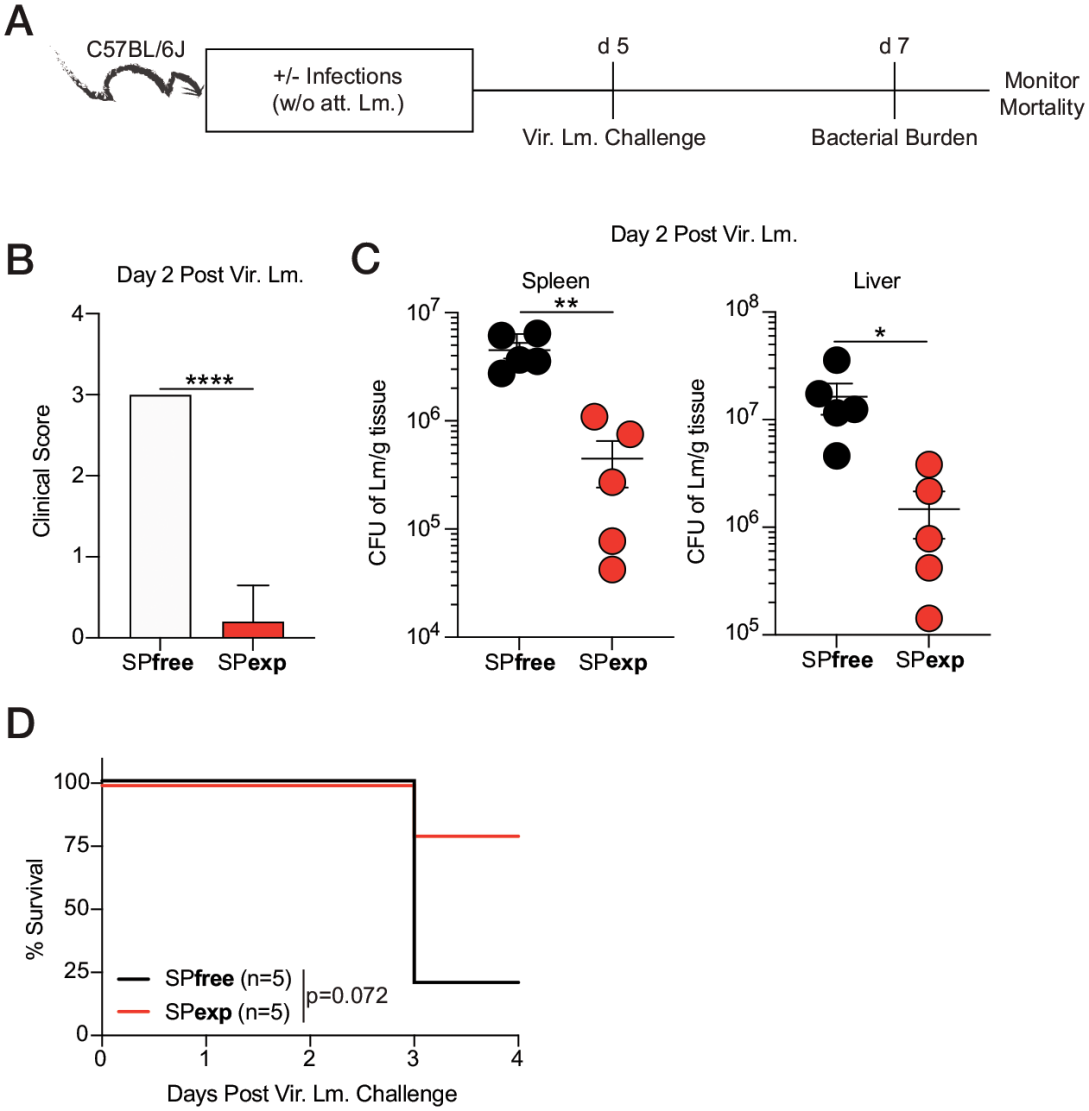
Enhanced control of virulent *L. monocytogenes* in SPexp mice. (**A**) Experimental design. SPfree and SPexp B6 mice (without attenuated *L. monocytogenes* infection) were challenged with 8 × 10^4^ CFUs virulent *L. monocytogenes* 5 d after last infection, and mortality was monitored. Bacterial burden was assessed within spleen and liver 2 d after virulent *L. monocytogenes* challenge. (**B** and **C**) Clinical score (B) and *L. monocytogenes* CFUs per gram of (left) spleen and (right) liver (C) 2 d after challenge. (**D**) Mortality of mice after challenge. Data are representative of at least three independent experiments with five mice per group. Error bars represent SEM. *p < 0.05, **p < 0.01, ****p < 0.0001.

The increased or decreased susceptibility observed in CoH mice relative to naive counterparts is dependent on the type of pathogen (16). To evaluate whether sequentially infected SPexp mice have pathogen-specific altered responses, we challenged SPexp and SPfree mice with virulent *L. monocytogenes* 5 d after the last infection. Bacterial clearance (spleen and liver) and morbidity/mortality were determined at indicated days after challenge (Fig. 4). Of note, the SPexp mice used in these experiments were generated without attenuated *L. monocytogenes* infection to prevent establishment of anti-*Listeria* immunity that would abrogate a productive *L. monocytogenes* infection (Fig. 4A). Interestingly, SPexp mice had decreased morbidity, assessed by clinical scoring, and decreased splenic and liver *Listeria* CFUs at day 2 after virulent *L. monocytogenes* infection (Fig. 4B, 4C). In addition, SPexp mice had increased survival after virulent *L. monocytogenes* infection, relative to naive SPfree controls (Fig. 4D).

Thus, the data in Figs. 3 and 4 collectively demonstrate that immunological experience achieved by sequential pathogen exposure differentially alters host responses to newly introduced infections. In addition, it recapitulates findings observed in CoH ‘dirty’ mice, suggesting the pathogens used here (type, dose, route, and order) could be used as an alternative to generate immunologically experienced hosts to expand the existing models available for addressing the translational gap present between studies based on SPfree mouse models and humans.

### Level of inflammation determines susceptibility to newly introduced infections in immune-experienced mice

To elucidate potential mechanism(s) that influence the susceptibility of SPexp (and CoH) mice to newly introduced infections, including sepsis, we focused on the basal inflammation present in immunologically experienced and/or naive mice. IFN-γ and TNF-α are key cytokines involved in the anti-Listerial immunity and clearance of *L. monocytogenes* (41–43), and both cytokines are increased in SPexp mice early after the last infection with LCMV-Arm (Fig. 2). In addition, elevated serum cytokines may contribute to the heightened cytokine storm in SPexp mice (Fig. 3); therefore, it is possible that the increased mortality to sepsis, but enhanced *L. monocytogenes* control, in SPexp mice is due to their heightened steady-state inflammation.

**FIGURE 5.**
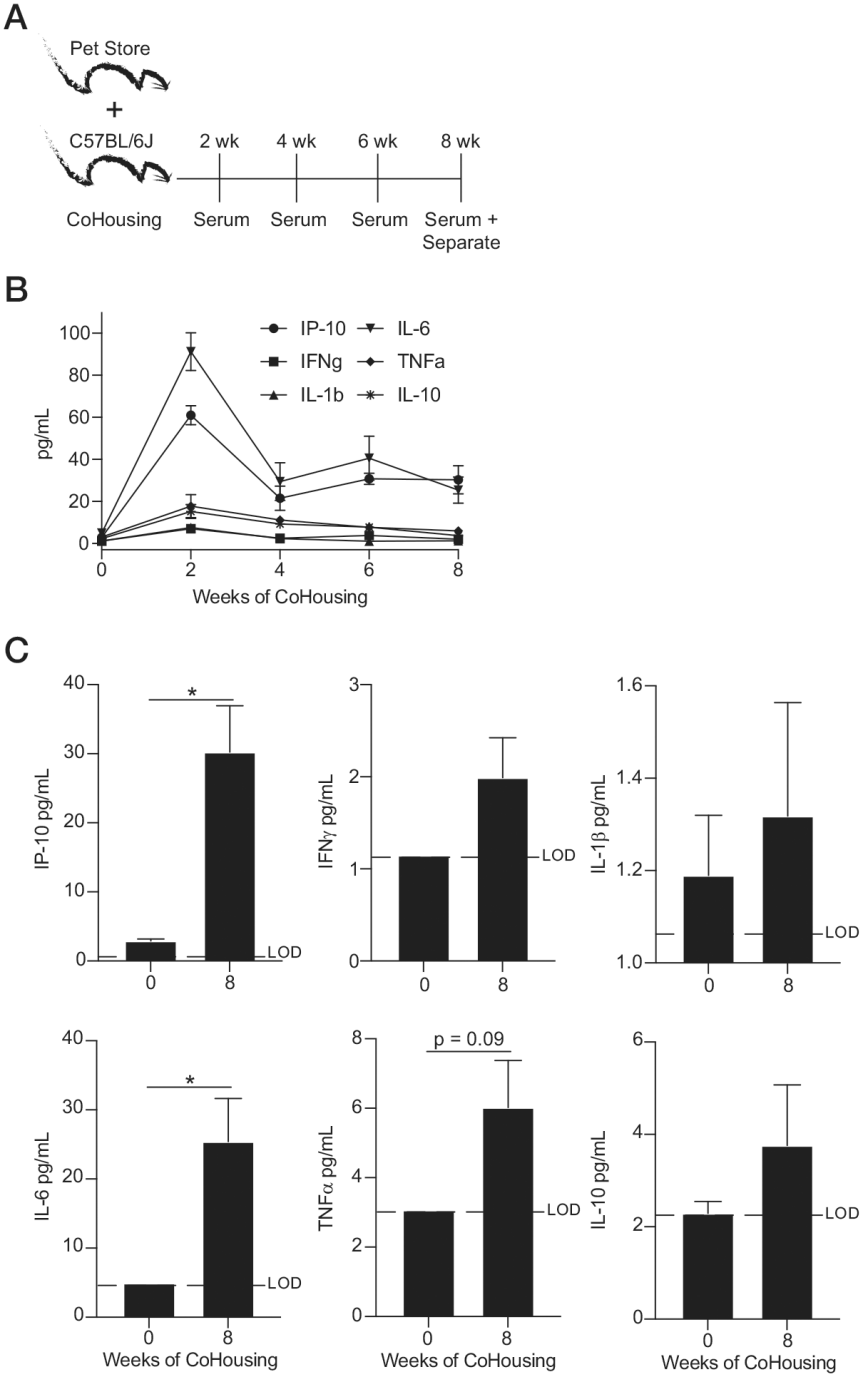
Prolonged inflammation in microbially experienced laboratory mice generated through cohousing with pet store mice. (**A**) Experimental design. One female pet store mouse was CoH with eight female B6 mice, and serum was collected at indicated times. (**B**) Longitudinal summary data of analyte levels throughout cohousing. (**C**) Analyte levels before and after 8 wk of cohousing. Data are representative of at least three independent experiments with 5–10 mice per group. Error bars represent SEM. *p < 0.05.

The contribution of heightened basal inflammation to subsequent infections observed in CoH mice cannot be easily interrogated because of the insufficient knowledge of the type of pathogens present/transmitted and timing of infection(s) (9). To define the duration of inflammation in CoH mice, we bled SPfree B6 mice before cohousing with pet store mice and then at weeks 2, 4, 6, and 8 of cohousing (Fig. 5A). Although all cytokines screened peaked after 2 wk of cohousing, the CoH mice still had heightened inflammation at 8 wk after initiation of the experiment (Fig. 5B, 5C). The cytokines/chemokines measured are involved in the control of *L. monocytogenes* and are present at high concentrations in the blood during the sepsis cytokine storm (16, 23, 33, 44, 45). Thus, long-lasting inflammation is observed in immune-experienced B6 mice generated by cohousing with pet store mice.

**FIGURE 6.**
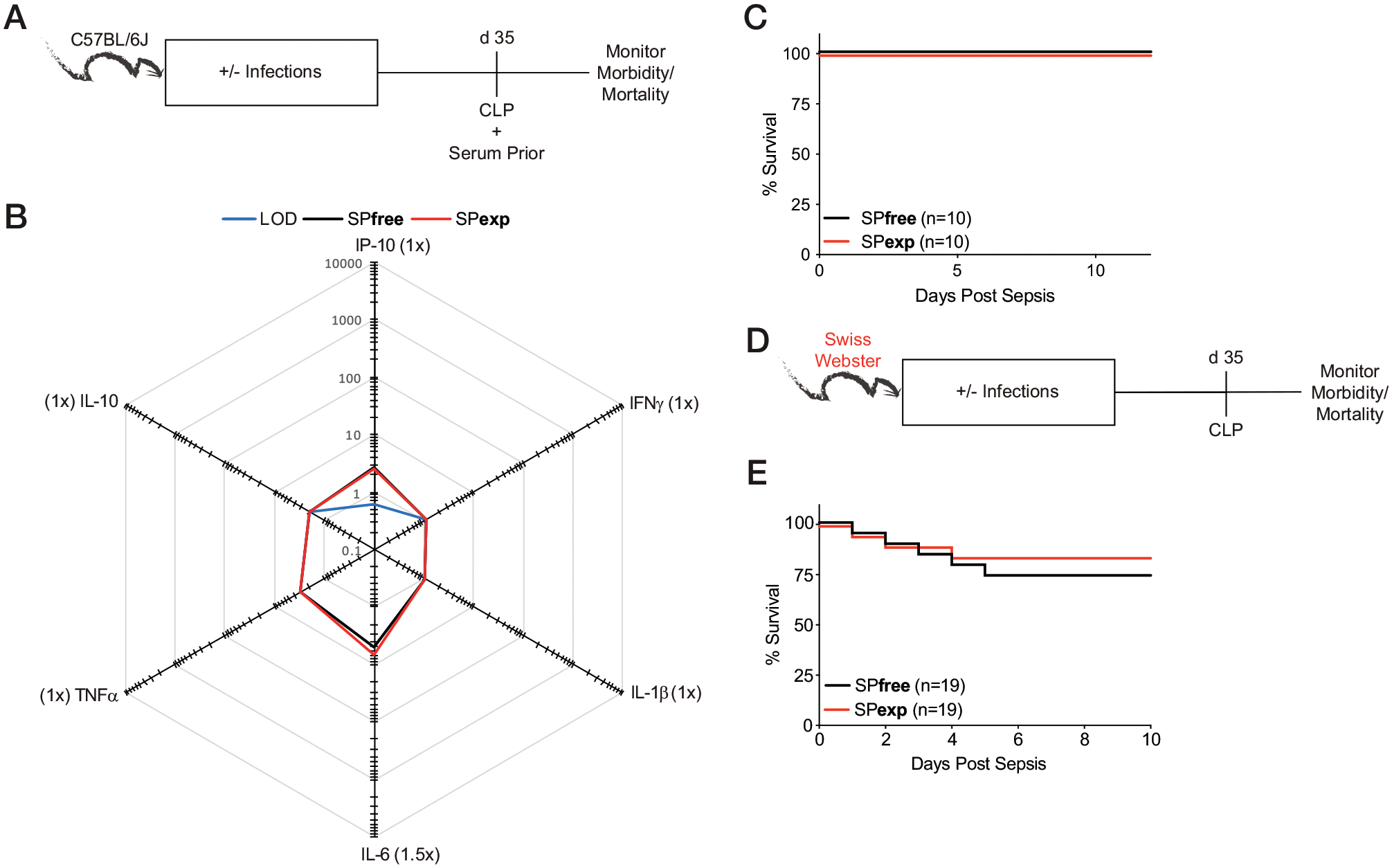
Susceptibility of immune-experienced hosts to sepsis correlates with the basal inflammation present at the time of challenge. (**A**) Experimental design. CLP was performed 35 d after last infection in SPexp and age-matched SPfree B6 mice. Body weight and mortality were monitored over time. Serum and PBLs were collected before CLP for baseline analyses. (**B**) Summary data of analyte concentrations (pg/ml) before CLP. Parentheses indicate fold difference between experimental groups. (**C**) Survival of mice after CLP. (**D**) Experimental design. Thirty-five days after last infection, CLP was induced in SPfree and SPexp Swiss Webster mice. (**E**) Survival of Swiss Webster mice after CLP. Data are representative of at least two independent experiments with 5–20 mice per group. Error bars represent SEM.

To test the contribution of the inflammatory milieu to differential responses observed in immunologically experienced mice, we returned to sequential infection model. Sera from SPfree and SPexp mice were evaluated before CLP performed 35 d after last infection (Fig. 6A). At this time point, SPfree and SPexp mice had equivalent basal inflammation (Fig 6B), demonstrating the SPexp mouse model enables us to “tune” the magnitude of inflammation before new infection. Importantly, when sepsis was induced 35 d after LCMV-Arm infection, the susceptibility of SPexp mice did not differ from SPfree B6 mice (Fig. 6C). Similar results were obtained in outbred Swiss Webster mice (Fig. 6D, 6E). The finding that immunological experience does not contribute to the increased susceptibility to sepsis at a time when basal inflammation is similar in both groups of mice was also confirmed when the severity of the septic insult was increased (Supplemental Fig. 2A–C).

**FIGURE 7.**
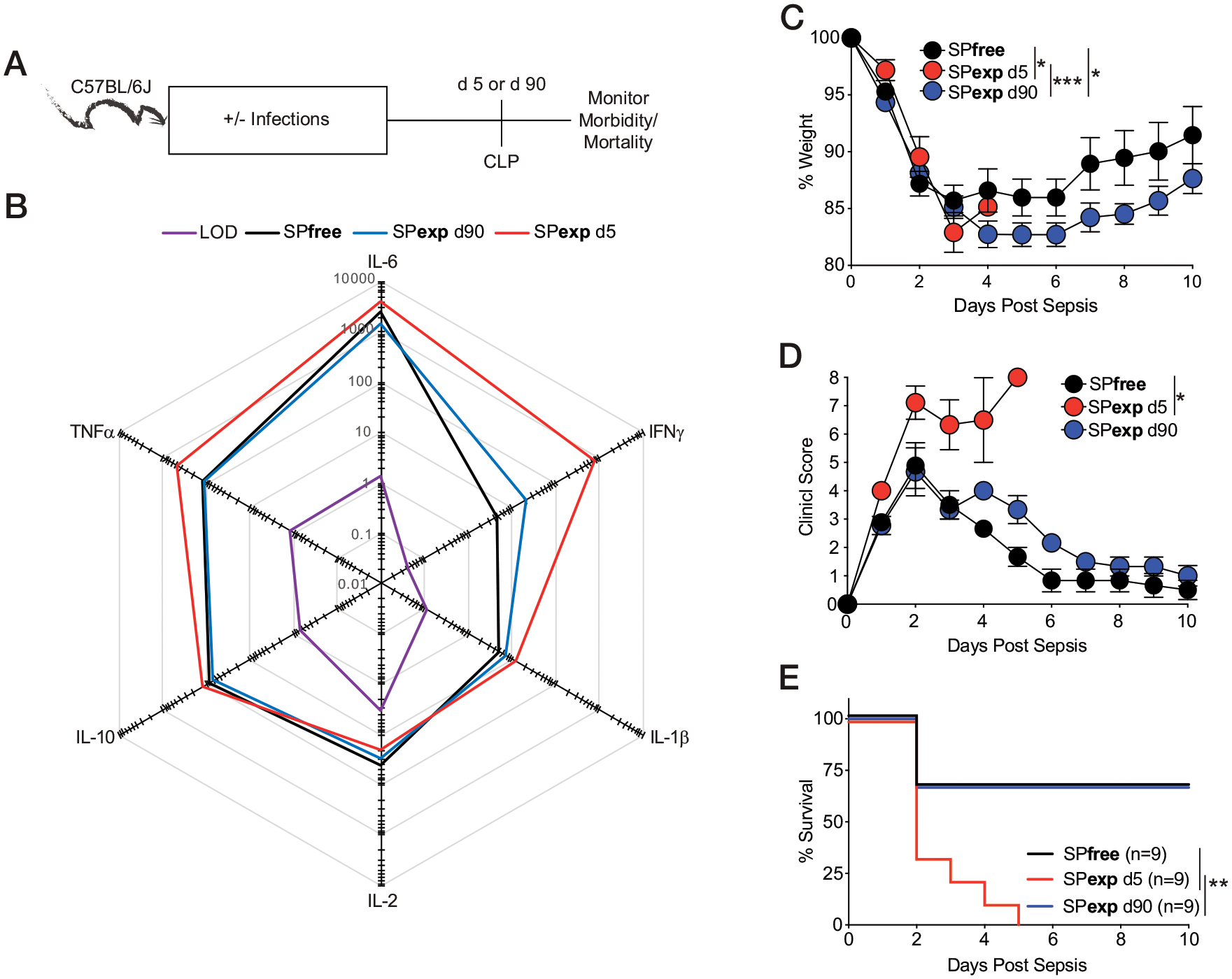
Heightened inflammation in SPexp mice contributes to increased mortality to CLP. (**A**) Experimental design. CLP was induced 5 or 90 d after last infection in SPexp B6 mice or in age-matched SPfree B6 mice. Body weight and mortality were monitored over time. Serum was collected before surgery. (**B**) Summary data of analyte concentrations (pg/ml) before CLP. (**C**–**E**) Weight loss (C), clinical score (D), and mortality (E) after CLP. Data are representative of at least two independent experiments with nine mice per group. Error bars represent SEM. *p < 0.05, **p < 0.01, ***p < 0.001.

In a complementary approach addressing the contribution of basal inflammation to sepsis susceptibility of SPexp mice, we waited 90 d after the last infection before performing CLP (Fig. 7). As a control, SPexp mice that had seen their last infection 5 d before CLP were included (SPexp day 5). As expected, SPexp day 5 mice had heightened cytokine storm inflammation, clinical score, and mortality after CLP, compared with SPfree and SPexp d90 mice (Fig. 7B–E). Importantly, SPfree and SPexp d90 mice had equivalent responses to CLP.

Finally, to establish whether enhanced anti-*Listerial* immunity in SPexp mice was dependent on the level of basal inflammation, we challenged SPexp mice with a high dose of virulent *L. monocytogenes* at day 5 or 35 after last infection together with SPfree mice (Supplemental Fig. 3A). Importantly, enhanced anti-*Listerial* immunity in SPexp day 5 mice (defined by the bacterial load in the spleen and liver 2 d after challenge or survival) was diminished in SPexp mice challenged with virulent *L. monocytogenes* 35 d after LCMV-Arm infection (Supplemental Fig. 3B, 3C).

Overall, these data suggest differential susceptibility of immune-experienced hosts to new infections (sepsis or *L. monocytogenes*) correlates with the basal inflammation (or cytokines/chemokines) present at the time of challenge.

### NK cells exacerbate mortality to sepsis in immune-experienced mice

**FIGURE 8.**
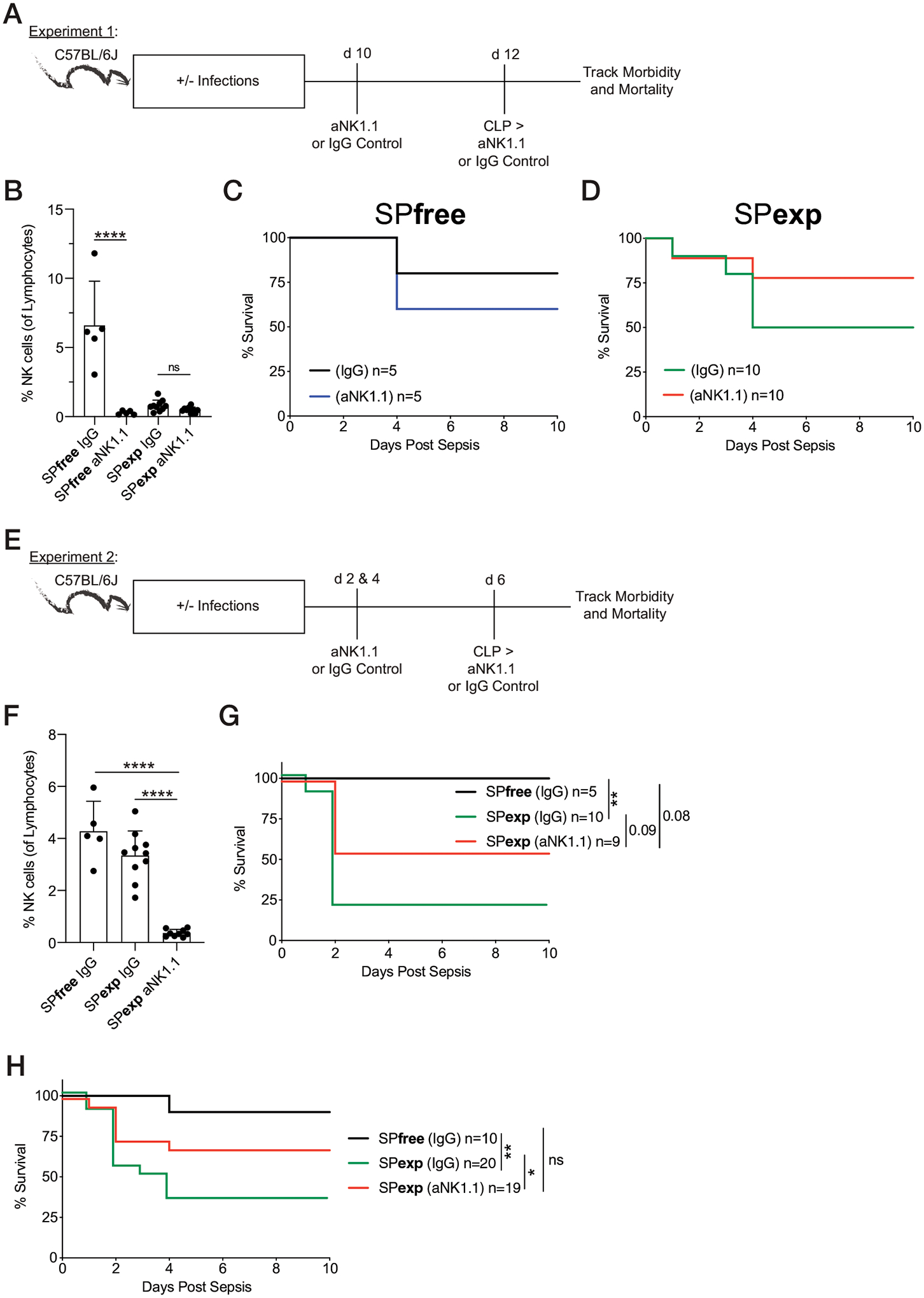
NK cells contribute to enhanced mortality of SPexp mice during septic event. (**A**) Experimental design. SPfree and SPexp B6 mice were given 300 mg of anti-NK1.1 mAb or control IgG 10 and 12 d after last infection. Day 12 administration of mAb was given after CLP procedure. (**B**) Frequencies of NK cells 12 h before CLP. (**C**) Survival of SPfree mice (control IgG and anti-NK1.1 mAb treated). (**D**) Survival of SPexp mice (control IgG and anti-NK1.1 mAb treated). (**E**) Experimental design. SPfree and SPexp mice were given 300 μg of anti-NK1.1 mAb or control IgG 2, 4, and 6 d after last infection. Day 6 administration of mAb was given after CLP procedure. (**F**) Frequencies of NK cells 12 h before CLP. (**G**) Survival of mice after CLP. (**H**) Experiments 1 and 2 combined survival after CLP. Data are representative of two independent experiments with 5–20 mice per group. Error bars represent SEM. *p < 0.05, **p < 0.01, ****p < 0.0001.

To further understand the immune changes responsible for enhanced mortality to CLP in immune-experienced mice, we next queried the contribution of NK cells to sepsis-induced mortality. This interest in NK cells comes from our recent data showing IFN-γ–producing NK cells can become IL-10 producers in response to the IL-15 produced during a septic event, and this NK cell–derived IL-10 is critical to support the survival of SPfree septic mice (23). NK cells contribute to the magnitude of both basal and cytokine storm inflammation (18, 23) and have the capacity to form memory populations in response to vaccination or infection (46), suggesting our immunological experience model has the ability to influence the functional capabilities and activation state of NK cells. MCMV-specific memory NK cells have increased accumulation and killing capacity after MCMV infection (46). Importantly, MCMV is a pathogen within our infection regimen, and IFN-γ, IL-10, and IL-15 are produced during the sepsis-associated cytokine storm (Fig. 2E) (23). To address the role of NK cell responses during sepsis in infection-experienced hosts, we administered anti-NK1.1–depleting mAb 10 and 12 d after the last infection (Fig. 8A). It is important to note that the day 12 dose of anti-NK1.1 mAb was administered immediately after CLP was performed. The frequency of NK cells among PBLs was significantly reduced in the anti-NK1.1 mAb–treated mice, relative to IgG controls, 12 h before septic induction (Fig. 8B). Consistent with our prior results (23), SPfree mice lacking NK cells had decreased survival after CLP (Fig. 8C). SPexp mice depleted of NK cells, in contrast, had enhanced survival to the septic event (Fig. 8D), suggesting NK cells foster unfavorable outcomes to sepsis in immune-experienced hosts. In a second experiment, we evaluated the contribution of NK cells to sepsis outcomes after three administrations of anti-NK1.1–depleting mAb, to induce an efficacious depletion strategy (Fig. 8E). Importantly, NK cells were now sufficiently depleted in SPexp mice, compared with IgG controls (Fig. 8F). Similar to observations in Fig. 8A–D, SPexp hosts lacking NK cells had enhanced survival relative to SPexp IgG-treated mice (Fig. 8G). In addition, when survival curves from both experiments are combined, SPexp mice deficient in NK cells demonstrated a significant increase in survival compared with SPexp IgG mice (Fig. 8H).

To unravel the mechanism behind enhanced survival of SPexp mice lacking NK cells, we next queried the extent to which depleting NK cells changed the inflammatory profile before and 12 h after CLP (Supplemental Fig. 4A, 4B, respectively). Out of the cytokines screened, NK cell–depleted SPexp mice had decreased levels of IL-10, IL-12p70, and IL-2 before and 12 h after septic insult (Supplemental Fig. 4A, 4B; respectively), suggesting NK cells contribute to the heightened inflammation observed in SPexp mice (before and after CLP). These data highlight how immunological experience, through sequential infection, alters NK cell status and responses that are detrimental to host survival during a septic event. Overall, NK-depleted SPexp mice have identified a cell type in part responsible for increased inflammation and susceptibility to sepsis in immune-experienced hosts. Lastly, these data elucidate a cell type to be therapeutically investigated in immune-experienced septic hosts, which was not revealed in conventionally used SPfree mice.

## DISCUSSION

Sepsis remains a significant disease that affects >125,000 people per day with ~20% succumbing to the septic event (3). The immune response elicited by sepsis is characterized by an initial transient cytokine storm composed of proinflammatory and anti-inflammatory cytokines, followed by a chronic state of immunoparalysis (44). To add to the complexity of the biphasic response during sepsis, close investigation of cytokine storm has shown it consists of two phases; within the first several hours proinflammatory cytokines are produced, which are then followed by anti-inflammatory cytokines as a counterbalance (44, 47). The cause of immunoparalysis is multifactorial, with decreased lymphocyte numbers, function, and changes in T cell repertoire (decreased T cell diversity) being some of the contributing features (48, 49). Lastly, the animal models used for preclinical interrogation often rely on SPfree mice that are immunologically more similar to a neonatal human, which may (inadvertently) exclude the large cohort of adult and elderly patients most commonly affected by sepsis. For these reasons many clinical trials targeting the cytokine storm have failed (4).

In this study, we demonstrate that in contrast with SPfree mice, the increased susceptibility of sequentially infected immunologically experienced (SPexp) mice to sepsis can be mitigated through NK cell depletion, which decreases the magnitude of basal and cytokine storm inflammation. The collective data presented in this article indicate our model of immune-experienced mice has the capacity to alter phenotype and function of various cellular compartments. Importantly, gene set enrichment analysis studies have shown that immune-experienced mice, generated by cohousing or sequential infection, have immune signatures similar to adult humans (12–14). Genes shared between experienced mice and adult humans are related to cytokine signaling, MHC regulation, and T cell activation. Our data, when combined with other studies using immune-experienced mice, further demonstrate the power of including immune-experienced mice in current preclinical research, and it is tempting to speculate on the benefit of reinterrogating the efficacy of current/new sepsis therapeutics in immune-experienced hosts.

To bridge the gap between mice and humans, several groups have used microbial exposure approaches in recent years to elicit an experienced immune status that is similar to adult humans. These approaches consist of sequential infection, cohousing with pet store mice, fomite (dirty bedding) exposure, and rewilding of previously naive mice (9, 10), with each containing their own strengths and weaknesses. In this study, we sequentially infected mice with five human-relevant laboratory pathogens to generate SPexp mice. This approach increased the number of Ag-experienced T cells and altered the activation status of cellular populations involved in a septic event, which was consistent with data observed in immune-experienced mice generated by cohousing and other models (16, 27, 28). Similarly, SPexp mice exhibited increased mortality after sepsis induction (Fig. 3), much like that seen for CoH mice (16), compared with SPfree mice. Notably, CoH mice have prolonged heightened basal inflammation lasting at least 8 wk (Fig. 5), making this model challenging to parse out the role of inflammation before a subsequent challenge. In contrast, the heightened inflammation in SPexp mice subsides by day 35 after last infection (Fig. 6), enabling investigation of the role of basal inflammation during a septic event in immune-experienced mice. Altering the infection regimen in the SPexp model also granted us the ability to demonstrate that the enhanced mortality in immune-experienced mice is due to heightened basal inflammation (Fig. 6). Changing infection regimens in cohousing, fomite, and rewilding models is unattainable due to uncontrollable environmental factors (7), suggesting our sequential infection model allows for the precise “tuning” of the immune experience status. In addition, pathogens transferred by natural infection are inconsistent due to the nature of the pathogen transfer (9), which we control for in SPexp mice by giving known doses of each pathogen. Another major hurdle when using nonsequentially infected experienced mice is the facility requirements, because some institutions may require BSL-3 level housing or an outdoor enclosure. Thus, facility requirements and the lack of controlled pathogen transfer creates increased financial burden for investigators, which can be mitigated by using SPexp mice housed under BSL-2 conditions. Overall, our SPexp murine model creates a defined immunologically experienced host with similar alterations observed in nonsequentially infected experienced models, and our model is accessible to a broader array of groups due to the reduced financial burden and potential housing limitations.

Although the magnitude and molecular composition of inflammation is defined by the activity of various immune cells, our data suggest NK cells are significant contributors to basal and sepsis-induced inflammation (Supplemental Fig. 4). In addition to their cytolytic capacity (46, 50), NK cells conventionally produce IFN-γ, but can produce IL-10 in response to IL-15 during sepsis (23). Interestingly, NK-depleted SPexp mice had decreased IL-10 without reducing IFN-γ, suggesting they primarily produce IL-10 in our model. Importantly, SPexp mice have increased survival when the systemic IL-10 concentration is decreased via NK cell depletion, advocating that IL-10 may be detrimental in immune-experienced mice, which is in direct contrast with SPfree mice (23). Notably, mice treated with exogenous IL-10 early after CLP surgery have increased mortality to the septic event (51). Furthermore, IL-10 blockade during in vitro stimulation of PBMCs enhanced IFN-γ and TNF-α production in PBMCs from septic patients, but not critically ill nonseptic patients or healthy controls (52). Therefore, proinflammatory cytokines produced during the septic event, but not at baseline, may be superior in supporting survival in immune-experienced hosts during a septic event, increasing the complexity of identifying therapeutic interventions for septic patients. Moreover, heightened basal inflammation is not only observed after sequential infection (or acute infections) but also during autoimmune settings, such as experimental autoimmune encephalomyelitis (EAE; murine model of multiple sclerosis). Importantly, Jensen et al. (53) show that EAE mice have increased mortality to CLP when EAE-induced inflammation is elevated; however, the cell type contributing to the elevated steady-state inflammation was not interrogated.

In summary, experimental data provided and discussed in this article emphasize the importance of interrogating immune-experienced models for preclinical studies to further define the cellular/molecular mechanism(s) that could be therapeutically exploited during severe and dysregulated infection-induced inflammatory responses, such as sepsis.

## Supplementary Material

1

## Abbreviations used in this article:

CLP: cecal ligation and puncture
CoH: cohoused
EAE: experimental autoimmune encephalomyelitis
IAV: influenza A virus
LCMV-Arm: lymphocytic choriomeningitis virus–Armstrong
MCMV: murine CMV
PBL: peripheral blood leukocyte
PR8: Puerto Rico/8/34
SPexp: specific pathogen experienced
SPfree: specific pathogen-free
