# Supplementary material for: Inflammation Controls Susceptibility of Immune-Experienced Mice to Sepsis
Source: Immunohorizons. Author manuscript; Available in PMC 2022 Nov 11. (PMC9650784; doi:10.4049/immunohorizons.2200050)
Supplement: 1 [file NIHMS1846481-supplement-1.pdf]

Supplemental Figure 1

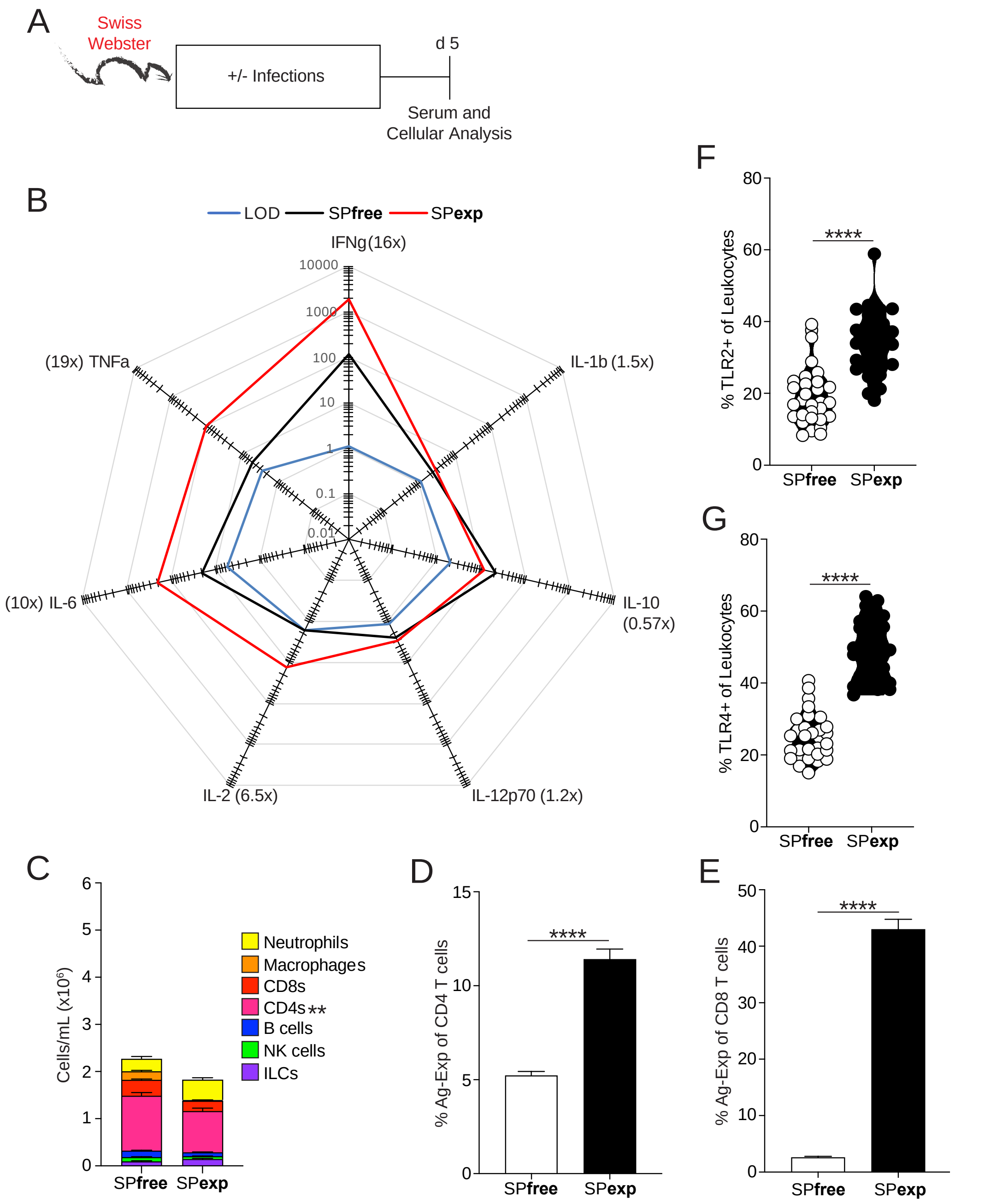

Supplemental Figure 1. Irrespective of host genetics, sequential infection leads to experienced immune status. A) Experimental design. Serum and PBL were collected from Swiss Webster SP**exp** mice, 5 d after last infection. **B**) Summary data of analyte concentrations (pg/mL) 5 d post last infection. **C**) Number of cells per mL of PBL, frequency of Ag-experienced **D**) CD4 and **E**) CD8 T cells, and **F**) frequency of TLR2- and **G**) TLR4-expressing leukocytes 5 days after last infection. Data are representatives from at least three independent experiments with 20 mice per group. Error bars represent SEM. **: $p < 0.01$, ****: $p < 0.0001$.

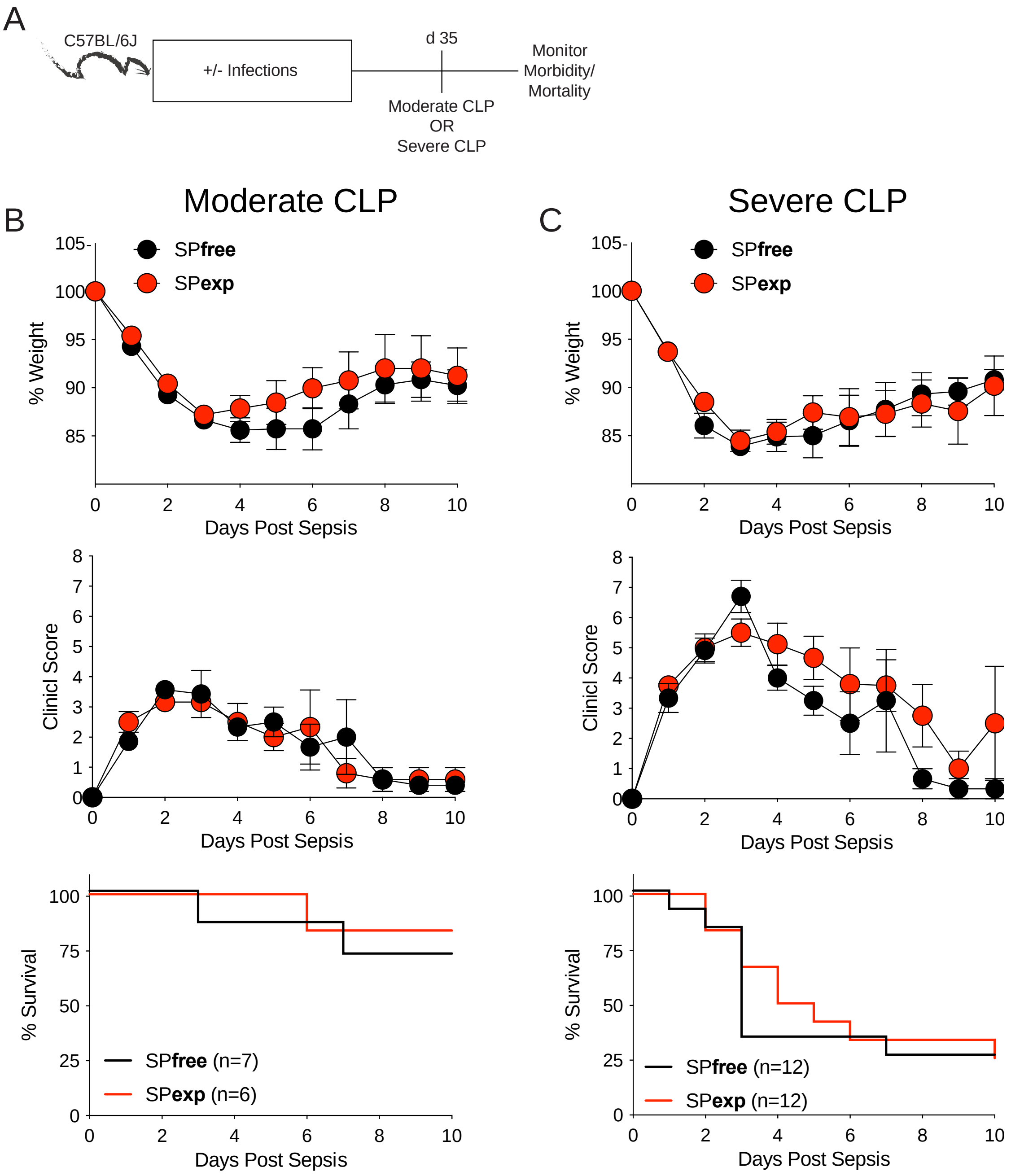

Supplemental Figure 2. Equivalent survival between SPfree and SPexp mice when severe septic event is 35 dpli. A) Experimental design. Moderate or severe CLP (~10-20% or ~50% mortality in SP**free** mice, respectively (23)) was performed 35 days post-last infection in SP**exp** and age-matched SP**free** B6 mice. Body weight and mortality were monitored over time. **B**) Weight, clinical score, and survival of mice following moderate CLP. **B**) Weight, clinical score, and survival of mice following severe CLP. Data are representatives from at least two independent experiments with 6-12 mice per group.

Supplemental Figure 3

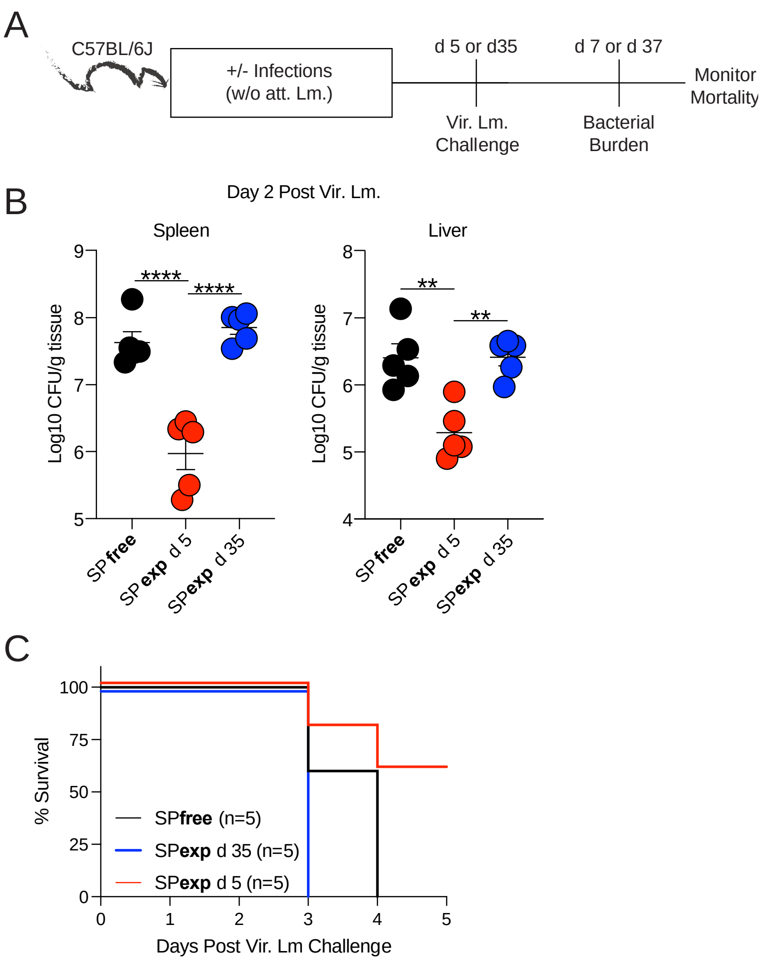

Supplemental Figure 3. Enhanced anti-Listerial response in immune-experienced hosts correlates with basal inflammation. A) Experimental design. SP**free** and SP**exp** mice (without attenuated *Lm.* infection) were challenged with 8 $\times$ 10^4 CFU virulent *Lm.* 5 or 35 d post last infection and mortality was monitored. Bacterial burden was assessed within spleen and liver 2 d after virulent *Lm.* challenge. **B**) *Lm.* CFU per gram of (left) spleen and (right) liver 2 d post challenge. **C**) Mortality of mice following challenge. Data are representatives from at least one independent experiments with 5 mice per group. Error bars represent SEM. **: $p < 0.01$, ****: $p < 0.0001$.

Supplemental Figure 4

A

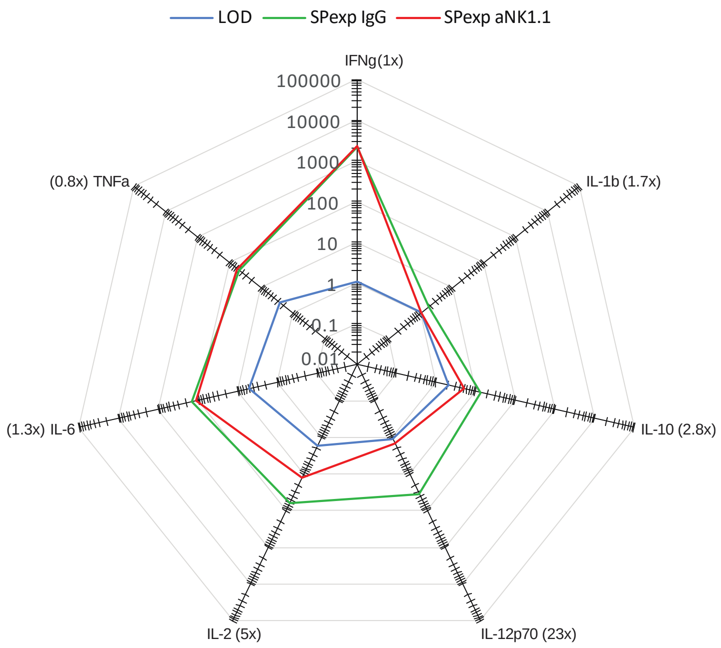

B

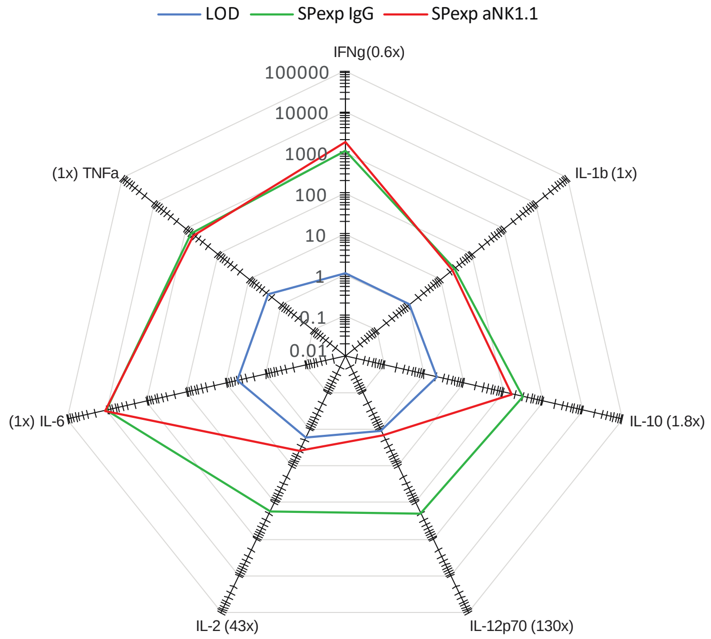

Supplemental Figure 4. NK cells contribute to basal and cytokine storm inflammation in SPexp mice. **A**) Summary data of analyte concentrations (pg/mL) 12 hr prior to CLP. **B**) Summary data of analyte levels 12 hr post CLP. Data are representatives from two independent experiments with 9-10 mice per group.
